# 1814. Acute Q fever: A 2-year Experience at a Tertiary-Care Center

**DOI:** 10.1093/ofid/ofac492.1444

**Published:** 2022-12-15

**Authors:** Said El Zein, Omar M Abu Saleh

**Affiliations:** Mayo Clinic, Rochester, Minnesota; Division of Public Health, Infectious Diseases, and Occupational Medicine, Department of Medicine, Mayo Clinic, Rochester, Minnesota

## Abstract

**Background:**

Diagnosing acute Q fever is challenging partly due to the non-specific and self-limited course of illness in the majority of cases. It is important to identify and treat patients who are at increased risk for progression to persistent disease to prevent future complications. We describe the demographics, clinical presentation, and treatment of patients diagnosed with acute Q fever at our institution.

**Methods:**

We identified all patients diagnosed with acute Q fever between December 2019 and March 2022. Patients 18 years or older who had a Q fever anti-phase II IgM ≥ 1:50 and anti-phase II IgG ≥ 200 were included. Patient with an anti-phase II IgG < 200 were included if they had a fourfold rise in titers at follow-up within 3-6 weeks.

**Results:**

Overall, 13 cases of acute Q fever were identified. Ages ranged between 32 and 68 years and 85% were men. The majority resided in the Midwest. A non-specific febrile illness was the most common presentation (11/13) (Table 1). Duration of symptoms predominantly ranged between 10 to 20 days. Elevated liver enzymes were noted in 7/13. Three had detectable Coxiella DNA in the blood during the acute phase using cell free DNA next generation sequencing but not using Coxiella PCR. Eleven patients had an exposure history. Overall, 6 patients received doxycycline monotherapy for a median of 14 days, while 7, who had risk factors for progression to chronic disease, received doxycycline and hydroxychloroquine for a median of 15 months. Among patients who followed-up, only one was restarted on therapy due to recurrence of symptoms. None of the patients developed infective endocarditis.

**Figure:**
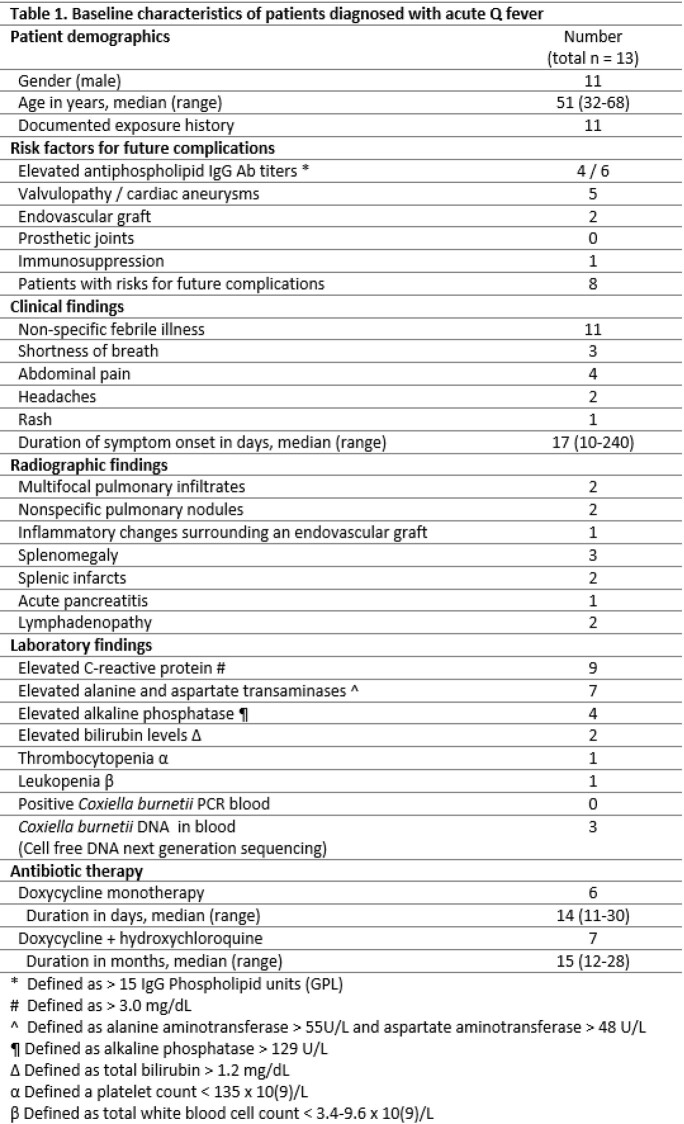

**Conclusion:**

Acute Q fever is more frequently recognized, partly, because of increased awareness among physicians, however also possibly due to the increasing prevalence of the infection. In a cohort of 49 patients with Q fever at our institution between 2012 and 2018, 20 had acute Q fever, compared to 13 patients in this cohort. The infection predominantly affects men and the majority have an identifiable exposure history. Elevated CRP, abnormal liver enzymes, and detectable antiphospholipid antibodies were prevalent in our cohort. Patient with risk factors for progression into chronic disease were often placed on a longer course of a combination therapy in attempt to prevent chronic focal infections.

**Disclosures:**

**All Authors**: No reported disclosures.

